# A CTLA-4 blocking strategy based on Nanoboby in dendritic cell-stimulated cytokine-induced killer cells enhances their anti-tumor effects

**DOI:** 10.1186/s12885-021-08732-5

**Published:** 2021-09-15

**Authors:** Wu Wang, Xi Wang, Wenli Yang, Kai Zhong, Na He, Xuexia Li, Yanyang Pang, Zi Lu, Aiqun Liu, Xiaoling Lu

**Affiliations:** 1 International Nanobody Research Center of Guangxi, College of Stomatology, Guangxi Medical University,Nanning, Guangxi, 530021 China; 2 Laboratory of Tropical Biomedicine and Biotechnology, School of Tropical Medicine and Laboratory Medicine, Hainan Medical University, Haikou, 570100 Hainan China; 3 Department of traditional Chinese medicine, The First Affiliated Hospital of Hainan Medical College, Haikou, 570100 Hainan China; 4 Department of Anesthesiology, Tunchang people’s Hospital, Tunchang, 571600 Hainan China; 5 Department of Anatomy, Zunyi Medical University, Zunyi, 563006 China; 6 Department of acupuncture and moxibustion, Hainan General Hospital, The Affiliated Hainan Hospitalof Hainan Medical University, Haikou, 570100 Hainan China; 7 Department of Laboratory Medicine, The second affiliated hospital of Hainan medical university, Haikou, 570311 Hainan China; 8 Affiliated Tumor Hospital, Guangxi Medical University, Nanning, 530021 Guangxi China

**Keywords:** Nanobody, Cytotoxic T-lymphocyte antigen-4, Cytokine-induced killer cells

## Abstract

**Background:**

Cytokine-induced killer cells induced with tumor antigen-pulsed dendritic cells (DC-CIK) immunotherapy is a promising strategy for the treatment of malignant tumors. However, itsefficacy isrestricted by the immunosuppression, which is mediated by the cytotoxic T-lymphocyte-associated antigen-4 (CTLA-4) pathway. In order to overcome the negative co-stimulation from these T cells,we screened a nanobody targeted for CTLA-4 (Nb36) and blocked the CTLA-4 signaling with Nb36.

**Methods:**

Peripheral blood mononuclear cells (PBMCs) were collected from healthy donors to beused to induce CIK cells in vitro, after which they were co-cultured with DC cells that had received tumor antigens. In addition, wetested whether blocking CTLA-4 signaling with Nb36 could promote in vitro DC-CIK cells proliferation, pro-inflammatory cytokine production and cytotoxicity,or not. For the in vivo experiments, we constructed a subcutaneously transplanted tumor model and placed it in NOD/SCID mice to verify the anti-tumor effect of this therapy.

**Results:**

After stimulation with Nb36, the DC-CIK cells presented enhanced proliferation and production of IFN-γ in vitro, which strengthened the killing effect on the tumor cells. For the in vivo experiments, it was found that Nb36-treated DC-CIK cells significantly inhibited the growth of subcutaneously transplanted livercancer tumors, as well as reduced the tumor weight and prolonged the survival of tumor-bearing NOD/SCID mice.

**Conclusions:**

Ourfindings demonstrated that in response to CTLA-4 specific nanobody stimulation, DC-CIK cells exhibited a better anti-tumor effect. In fact, this Nb-based CTLA-4 blocking strategy achieved an anti-tumor efficacy close to that of monoclonal antibodies. Our findings suggest that DC-CIK cells + Nb36 have the potential totreatmalignant tumors through in vivo adoptive therapy.

## Background

Cancer immunotherapy obtainsbeneficial effects bymediating tumor cell regression, which relies on the activation, persistence and targeting of anti-tumor T-cells [1]. Cytokine-induced killer cells (CIK) are immunologically active cells that have beenexpanded in vitro, and which haveboth the strong cytotoxicity of T-lymphocytes and the non-MHC-restricted killing characteristics of NK cells [2]. As the most powerful antigen-presenting cells, dendritic cells (DCs) can capture the antigen and then present it to the surface of a responder (such as CIK), thereby activating the antigen-specific immune response and improving the function of the effector cells. DC-CIK therapy has the potential for both MHC-unrestricted and tumor-specific activity [3]. However, during solid tumor therapy,it wasfound that an immunosuppression of the tumor’s microenvironmentled to the restriction ofthe cytotoxicity and proliferation of DC-CIKs in vivo [4]. In addition to dysfunctional stromal cells in the tumor microenvironment, there are also various regulatory T-cells, myeloid-derived suppressor cells, and up-regulated tumor suppressor molecules such as cytotoxic T-lymphocyte-associated antigen-4 (CTLA-4) and programmed cell death protein-1 (PD-1) [5, 6]. This complex tumor microenvironment is a hotbed for tumor developmentand leads to the exhaustion of infused DC-CIK cells, and is thereforethe main mechanism limiting the efficacy of DC-CIK adoptive therapy [7].

CTLA-4 is a protein receptor mainly expressed in both activated and regulatory T-cells. CTLA-4 competes with CD28 for ligand binding and emits inhibitory signals to attenuate T-cell activation [8]. Expressions of CTLA-4 areusually up-regulated with the continuous activation of T-cells. CTLA-4 can be combined with molecules of the B7 family on the DCs’ surface as a co-stimulatory signal to inhibit the proliferation, activation and cell cycle of the T-cells. Thisleads to decreased secretion of cytokines such as IL-2, IL-4, IFN-γ and decreased expression of IL-2 receptors. This opens a window for tumors to escape fromimmune surveillance by negatively regulating T-cellproliferation [8–10]. As one of the most important immunosuppressive receptors, CTLA-4-targeted immune checkpoint inhibitors are also an important and much researchedtopic in tumor immunotherapy. The blockade of the CTLA-4 signaling with monoclonal antibodies leads to an enhancement of the antitumor immune response, meaning that it has become a potential tumor immunotherapy strategy and is currently undergoing a number of clinical investigations [11, 12]. However, this strategy presentsmany limitations, including the non-specific binding of monoclonal antibodies to normal tissue and heterogeneous tumor antigens, and poor penetration of antibodies in the tumor microenvironment. Therefore, finding a way to develop novel antibodies with high efficiency and low toxicity is a matter of great urgency [13].

**Fig. 1:**
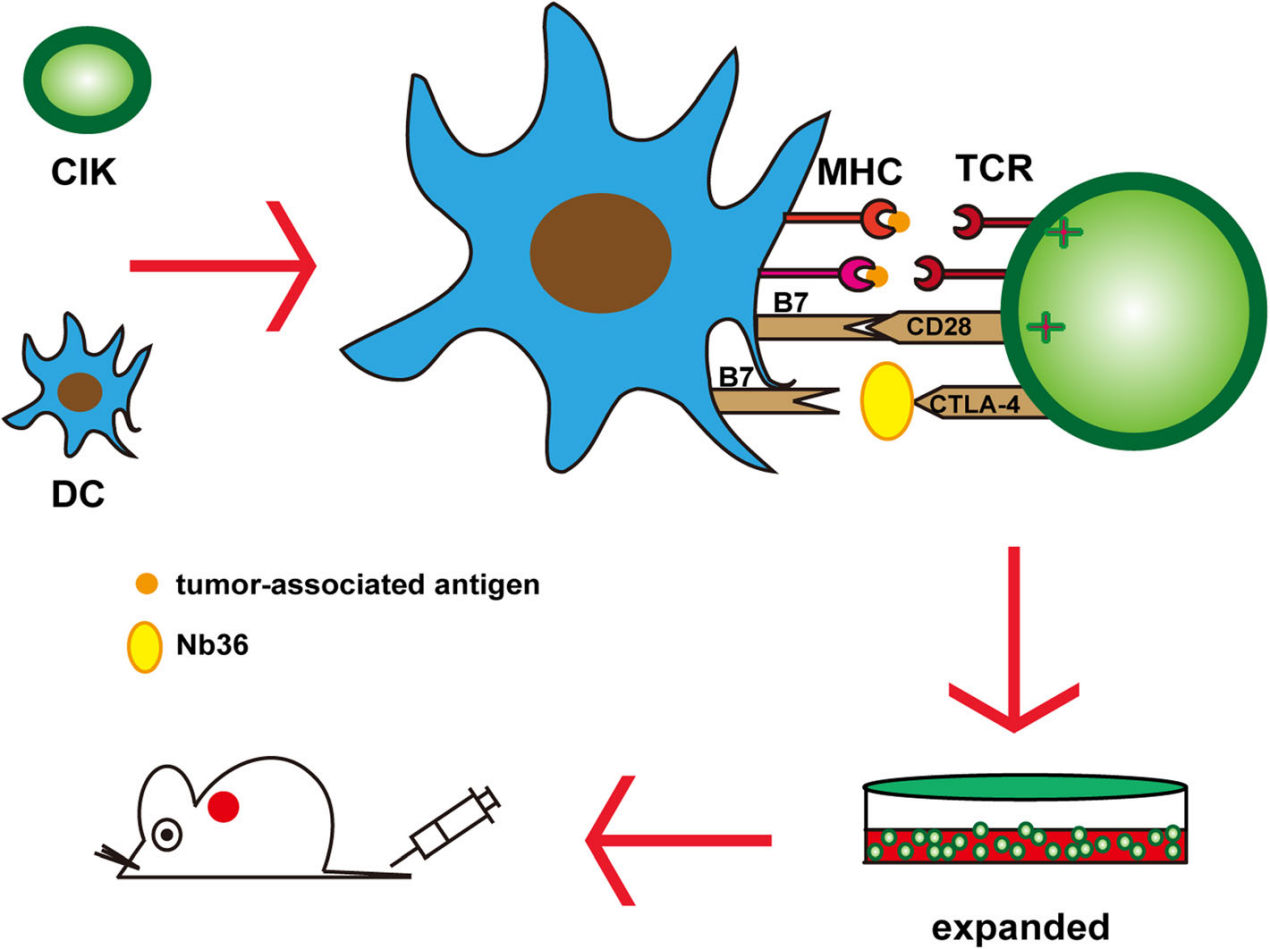
Therapy strategy with DC-CIK cells+anti-CTLA-4 nanobodies. DC-CIK cells were first generated. Then, the nanobodies against CTLA-4 (Nb36) eliminated immunosuppression via blocking CTLA-4-mediated negative co-stimulation in the effector DC-CIK cells. Finally, DC-CIK cells were transferred to kill the tumor cells

A nanobody (Nb) is a special single domain antibody which is derived from camelids. These animals naturally possess a special single-chain antibody (lacking light chain and CH1), and Nb is obtained after cloning its variable regions; which is in fact the smallest antigen-binding unit ever found [14]. Its simple molecular structure allows Nb to bind to epitopes that are not easily accessible to traditional antibodies [15]. In addition, Nb has a strong tolerance to changes in temperature and pH, and furthermore, its stable conformation allows it to be taken orally. Compared with conventional antibodies, Nb has high specificity, good physical and chemical stability, high yield, low cost, and lacks immunogenicity, which make it very suitable toaclinical setting [16]. In previous studies, we used phage display technology to obtain a set of CTLA-4 specific Nb. This anti-CTLA-4 Nb (Nb36) can bind to CTLA-4 epitopes on the surface of activated T-cells in vitro [17, 18]. To further investigate its antitumor activity in this study, we have used these novel antibodies to block CTLA-4 signaling in activated DC-CIK cells, thereby promoting the ability of DC-CIK to survive, proliferate and infiltrate (Fig. 1).

## Materials and methods

### Animals and cell culture

Female NOD/SCID mice, aged 4–6 weeks, were purchased from Beijing Vital River Lab of Animal Technology (Beijing, China) and raised in anSPF environment. All protocols were approved by the Animal Ethics Committee of Hainan Medical University. HepG2 (hepatoma carcinoma) and A549 cells (lung carcinoma) were purchased from the International Nanobody Research Center of Guangxi and cultured in DMEM supplied with 10% fetal bovine serum (FBS) and penicillin/streptomycin in 37 °C, 5% CO_2_.

### Antibodies and Nanobodies

The anti-CTLA-4 Nb (Nb36) wasdeveloped in our previous study [17]. Anti-CTLA-4 mAb (Rabbit monoclonal, UC10-4F10–11), anti-CD3 mAb (Rabbit monoclonal, SP7), anti-CD56 mAb (Rabbit monoclonal, EPR21827), anti-CD80 mAb (Rabbit monoclonal, EPR1157(2)), anti-CD83 mAb (Rabbit monoclonal, EPR23809–19) and anti-MHC II mAb (Rabbit monoclonal, 6C6) were all purchased from Abcam (Cambridge, UK).

### Generation of CIK and DCs

Density gradient centrifugation was used to collectperipheral blood mononuclear cells (PBMCs) from healthy donors, after which they werecultured in Roswell Park Memorial Institute (RPMI)-1640 medium (Gibco), containing 10% FBS and penicillin/streptomycin. Following 4 h of culture, the suspended cells (T-cells) were grown to generate CIK cells in RPMI-1640 with 10% FBS, containing 500 ng/ml anti-CD3 antibody, 100 U/ml IFN-γ (Servicebio, Wuhan, China) and 10 μg/ml polyhydroxyalkanoates (Solarbio, Beijing, China). The cell concentration was adjusted to1 × 10^6^ cells/ml. In addition, the adhered cells (1 × 10^5^ cells/ml) were used for dendritic cell differentiation via culturing in RPMI-1640(containing 10% FBS and penicillin/streptomycin), supplemented with 1000 U/ml recombinant human GM-CSF (rhGM-CSF; R&D, MN, US) and 500 U/ml rhIL-4 (R&D, MN, US) for 7 days. Next, A549 and HepG2 cells were lysed (repeated freeze-thaw procedure), andthe supernatant was obtained as the tumor antigen. On day 8, the supernatant as well as10 ng/ml TNF-αand 10 ng/ml IL-1β were added to the DCs’ medium, after which the culture was maintained for 2 days. On day 10, DCs and CIKs were co-cultured at a ratio of 1:10 for 2 days (RPMI-1640 with 10% FBS containing 500 ng/ml anti-CD3 antibodies). On day 12, we added the Nb36 to the DC-CIK cells to mediate the CTLA-4 blockade [19]. This study has beenapproved by the local ethics committee of Hainan Medical University.

### Phenotypic analysis of CIK and DCs

On day 7, flow cytometry was used to analyze the HLA-DR, CD80 and CD83 expression of DCs. Following this, a phenotypic analysis of DC-CIK cells, including CD56 and CD3 was performed by flow cytometry on day 14(Backman CytoFlex S).

### Cell proliferation assay

In vitroCFSE staining was used to determine the proliferation of DC-CIK cells following theirstimulation by tumor antigens. Individual groups of DC-CIK cells were stimulated with Nb36 (50 μg/ml), anti-CTLA-4 mAb (50 μg/ml), and anti-CD105 Nb (Isotype Control, 50 μg/ml) (1 × 10^6^ cells/tube), after which they werelabeled with CFSE (Sigma-Aldrich, MO, US) at 37 °C for 5 min. After washing with PBS, the cells were co-cultured with the same number of HepG2 cells that were exposed with a cell irradiator to 100 Gy of radiation for 120 h. The suspended CIK cells were harvested, and the percentage of proliferative CIK cells was determined by flow cytometry (Backman CytoFlex S).

### Cytotoxicity assay

We next assessed the cytotoxicity of DC-CIK cells against HepG2 and A549 cells in vitro. After being stimulated with Nb36, anti-CTLA-4 mAb, or anti-CD105 Nb, the DC-CIK cells were collected and mixed with HepG2, or with A549 cells with E:T ratios of 5:1, 10:1 or 20:1. The target cells with PKH26 staining were co-cultured for 6 h at 37 °C, 5% CO_2_. The PKH26-labeled cells were stained with propidium iodide (PI) and flow cytometry was used to determine the percentage of PKH26^+^PI^+^ dead cells (Backman CytoFlex S).

### ELISA and ELISPOT assays

DC-CIK cells and T-cells (control, 1 × 10^5^/well) were co-cultured with HepG2 cells (1 × 10^5^/well) for 16 h. We used ELISA to detect the supernatant’slevels of IL-2, TNF-αand IL-10. Meanwhile, ELISPOT assay was performed to detect the ratio of effector cells which specifically secreted IFN-γ. Briefly, DC-CIK cells and T-cells (control, 3 × 10^5^/well) were co-cultured in triplicate with the HepG2 cells (1 × 10^5^/well) that had been irradiated in plates precoated with anti-IFN-γat 37 °C for 12 h. After being washedwith PBS, biotinylated anti-IFN-γwere added and reacted at 4 °C for 12 h. Then, the IFN-γ specific immunocomplex wasanalyzed by streptavidin ap and observed in the substrate solution (BCIP/NBT). The number of spotswasdetected using a CTL ImmunoSpot S6 Ultimate-V analyzer.

### Xenograft experiments in mice

For xenograft experiments, each NOD/SCID mousewas implanted with HepG2 cells (2 × 10^6^/injection) via subcutaneous injection in the left armpit. Once the tumors reached a volume of 100 mm^3^, these tumor-bearing mice were injectedthree times every 7 days through the tail vein with 5 × 10^6^ DC-CIK cells, Nb36-treated DC-CIK cells, anti-CTLA-4 mAb-treated DC-CIK cells, anti-CD105 Nb-treated DC-CIK cells, or PBS. The tumor volume was then recorded with a vernier caliper every 3 days to evaluate tumor growth. Tumor volume (V) was calculated according to the formula: V = 0.5ab^2^, where a is the largest diameter, and b is the perpendicular diameter.

### Immunohistochemistry

The tumor tissue samples were fixed with 10% neutral formalin before paraffin embedding, and the slice thickness was 4-μm. Next, these sections were incubated with anti-ki67 monoclonal antibodies overnight at 4 °C. After washing twice, the sections were further incubated with the HRP-labeled secondary antibodies. Images were obtained with a microscope (Nikon, Japan).

### Statistical analysis

The FACS data was analyzed using FlowJo v10.07 software, while GraphPad Prism software 6.0 was utilized for the statistical analysis of data. The differences found between the experiments’different groups of data wereanalyzed using One-way ANOVA alongsideTukey’s multiple comparison. The tumor growth curve was evaluated using two-way ANOVA with corrections for Tukey’s multiple comparison. The survival curve for themice in the different groups was analyzed using Kaplan-Meier analysis (log-rank test).

## Results

### Characterization of the Nb36-treated DC-CIK cells

**Fig. 2:**
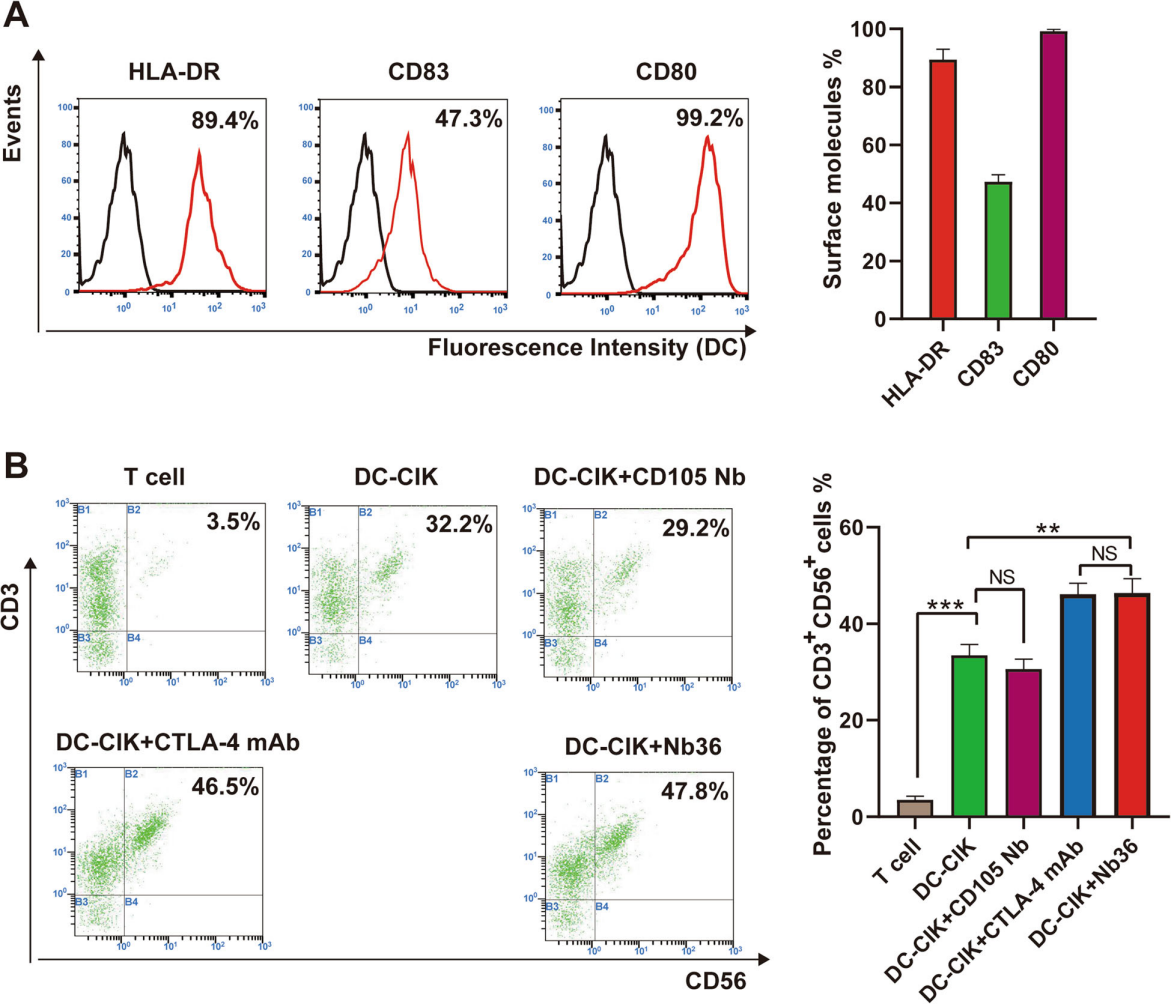
Main DC-CIK cellphenotypes derived from donors. **A** On day 7, DCs exhibited HLA-DR expression and co-stimulatory molecules (CD83 and CD80) in FCs. **B** DC-CIK cells in different groups exhibited CD3/CD56 expression in FCs. *n* = 3, **P < 0.01, *** *P* < 0.001. FlowJo v10.07 software was utilized for statistical analysis

Flow cytometry was used to monitor the immunophenotype of the ex vivo cultivated cells. The high expression of HLA-DR (89.4 ± 3.55%), co-stimulatory molecules CD80 (99.2 ± 0.7%), and the maturation marker CD83 (47.3 ± 2.38%) found on day 7 demonstrated the maturation of the DCs (Fig. 2A). These DCs were stimulated by tumor antigens and incubated with CIK. The CIK cells had the CD3^+^CD56^+^ phenotype in the DC-CIK + Nb36 group with a total cells median percentage of 46.43% (range 43.1–48.4%), which was significantly higher than the untreated DC-CIK group (33.46%, range 32.1–36.1%) and close to the CTLA-4 mAb treated group (46.16%, range 42.8–49.42%) (Fig. 2B). These results indicate that DC-CIK cells have a more efficient activation and expansion when CTLA-4 signaling isblocked.

### Blocking CTLA-4 signaling with Nb36 promoted proliferation of DC-CIK cells in vitro

**Fig. 3:**
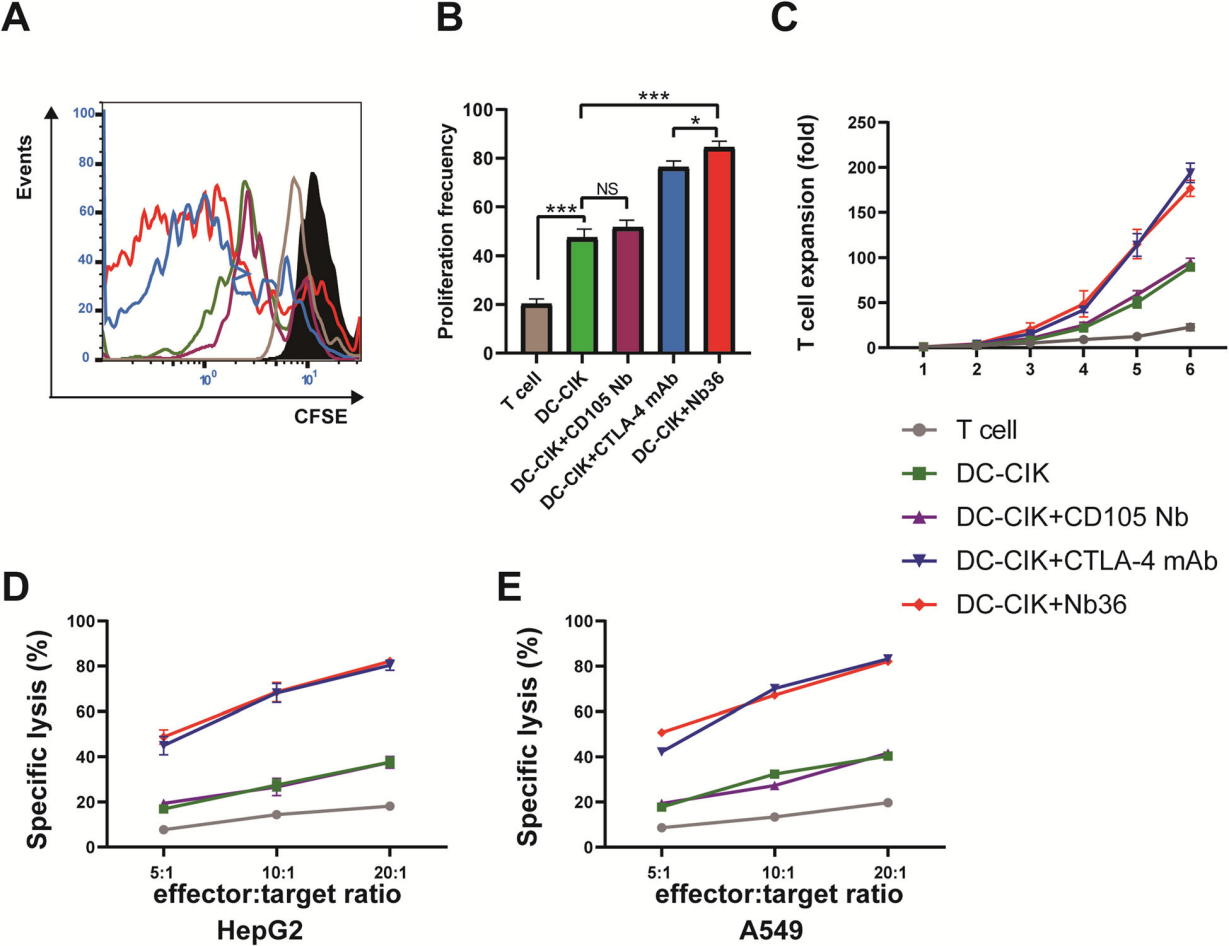
Nb36 promoted DC-CIK cell proliferation and eliminated target cells. **A** CFSE-labeled DC-CIK cells were mixed with different antibodies and co-cultured for 120 h. DC-CIKcell proliferation was assessed via flow cytometry. **B** Quantitative analysis of proliferation frequency of DC-CIK cells. *n* = 3, *P < 0.05, *** *P* < 0.001. **C** Expansion folds of DC-CIK cells stimulated with HepG2. *n* = 3. **D** and **E** DC-CIK cells were co-incubated with the indicated cells, and labeled with PKH26 at the E/T ratios of 5:1, 10:1 and 20:1 for 6 h. The ratios of PHK26^+^PI^+^ were measured by flow cytometry. Nb36-treated DC-CIK cells had enhanced cytotoxicity against HepG2 and A549 target cells. *n* = 3.FlowJo v10.07 software was utilized for statistical analysis

We tested whether blocking CTLA-4 signaling with anti-CTLA-4 Nb could promote DC-CIK cells proliferation and pro-inflammatory cytokine production in vitro. Theresults showed that 120 h ofstimulation with tumor cells (HepG2) significantly promoted the proliferation of CFSE-labeled DC-CIK cells in the DC-CIK + Nb36 group, promoting it far more than in the DC-CIK (*p* < 0.001) and DC-CIK + CTLA-4 mAb (*p* < 0.05) groups (Fig. 3A-B). In addition, longitudinal measurements of Nb36-treated DC-CIK cells proliferation suggested that the number of CFSE-labeled DC-CIK cells was significantly greater than in other groups, except for in the DC-CIK + CTLA-4 mAb group. After 11 days of cultivation, the number of DC-CIK cells in the DC-CIK + Nb36 group had increased by 180 times (Fig. 3C). These data therefore indicate that blocking CTLA-4 signaling with Nb36 further activates DC-CIK cells and promotes cell proliferation in vitro.

### Blocking CTLA-4 signaling with Nb36 facilitated the tumor cell killing of DC-CIK cells in vitro

A cytotoxicity assay was performed to examine theimpact Nb36-treated DC-CIK cells had on target cell killing. The results indicated that the effector-target ratios of (E:T) 5:1, 10:1 or 20:1had a much higher killing effect on HepG2 and A549 cellsfor the effector cells in the DC-CIK + Nb36 group than for theT-cell, DC-CIK and DC-CIK + CD105 Nb cells, with thesehavinglow or little cytotoxicity against such target cells (Fig. 3D-E). Hence, cells in the DC-CIK + Nb36 group were shown to effectively and specifically kill target cells in vitro, with theirefficiency beingclose to the DC-CIK + CTLA-4 mAb group.

### Blocking CTLA-4 signaling with Nb36 stimulated DC-CIK cells activation and pro-inflammatory cytokine production in vitro

**Fig. 4:**
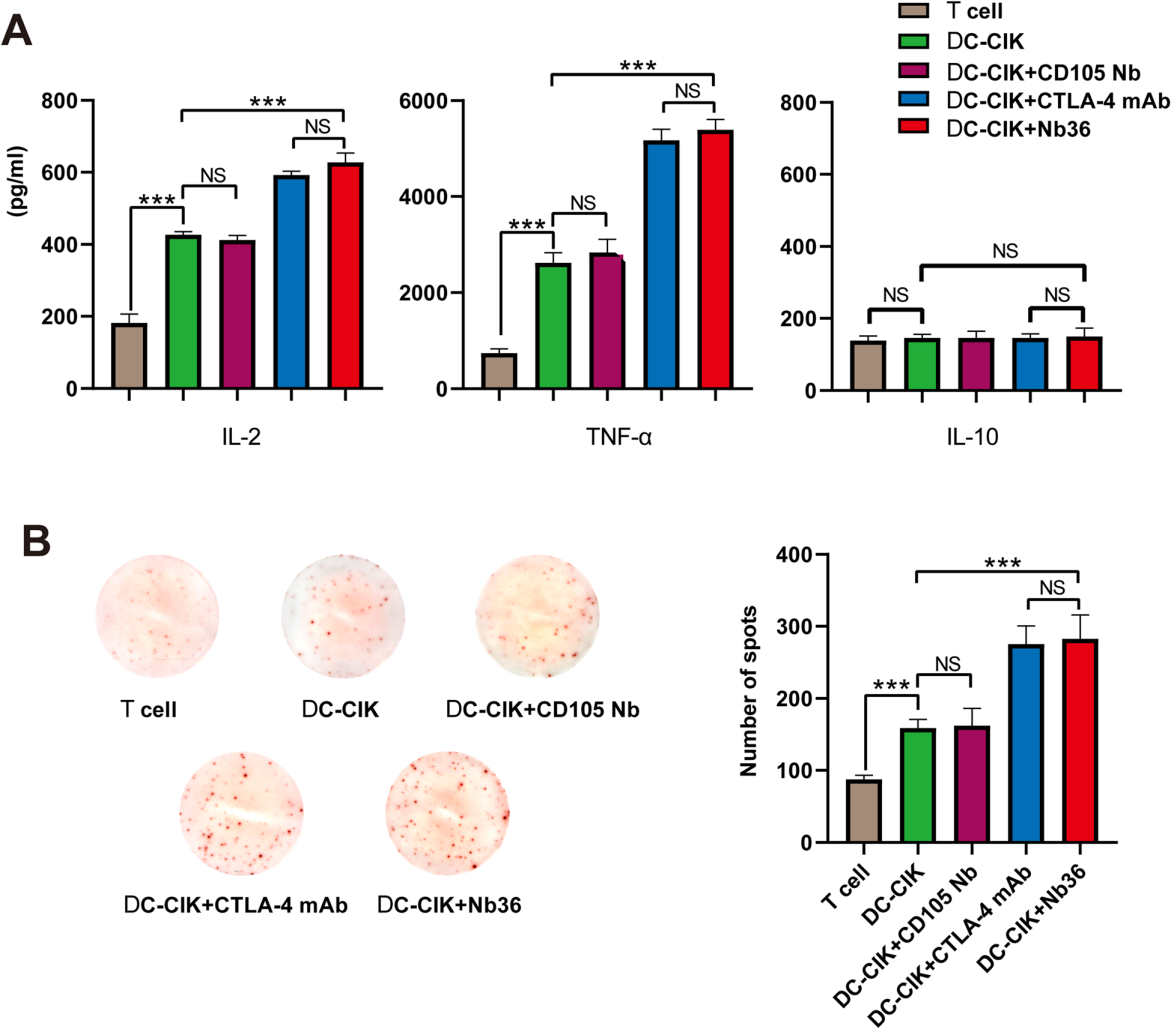
Nb36 increased the abundance of IFN-γ-secreting effector DC-CIK cells. **A** Nb36-treated DC-CIK cells produced higher levels of pro-inflammatory cytokines viz. TNF-α and IL-2, except IL-10. DC-CIK cells were incubated with tumor cells for 16 h, and the levels of pro-inflammatory factors in the supernatants were determined by ELISA. **B** ELISPOT analysis of the frequency of IFN-γ-secreted DC-CIK cells. *n* = 3, *** *P* < 0.001

We tested whether blocking CTLA-4 signaling with Nb36 could stimulateDC-CIK cells activation and pro-inflammatory cytokine production in vitro. To test this, groups of DC-CIK cells were stimulated with the same number of HepG2 cells for 12 h. Then, the supernatants’ levels of IL-2, TNF-α and IL-10 were measured by ELISA. We found that after being challenged with target cells,the levels of IL-2 and TNF-α in the supernatants belonging tothe cultured Nb36-treated DC-CIK group were significantly higher than those in the DC-CIK and anti-CD105 Nb-treated DC-CIK groups (Fig. 4A). However, no significant difference could be seen in the expression of IL-10 among the different groups (Fig. 4A). ELISPOT revealed that after being challenged with HepG2 cells,the number of IFN-γ-secreting spot forming cells in the Nb36-treated DC-CIK group was significantly greater than those found in the T-cells, DC-CIK cells and DC-CIK + CD105 Nb groups (Fig. 4B). These data therefore demonstrate that Nb36-treated DC-CIK cells have activated and secrete higher levels of pro-inflammatory cytokines after being challenged with target cells.

### Nb36 increased the anti-tumor efficacy of DC-CIK cells in vivo

**Fig. 5:**
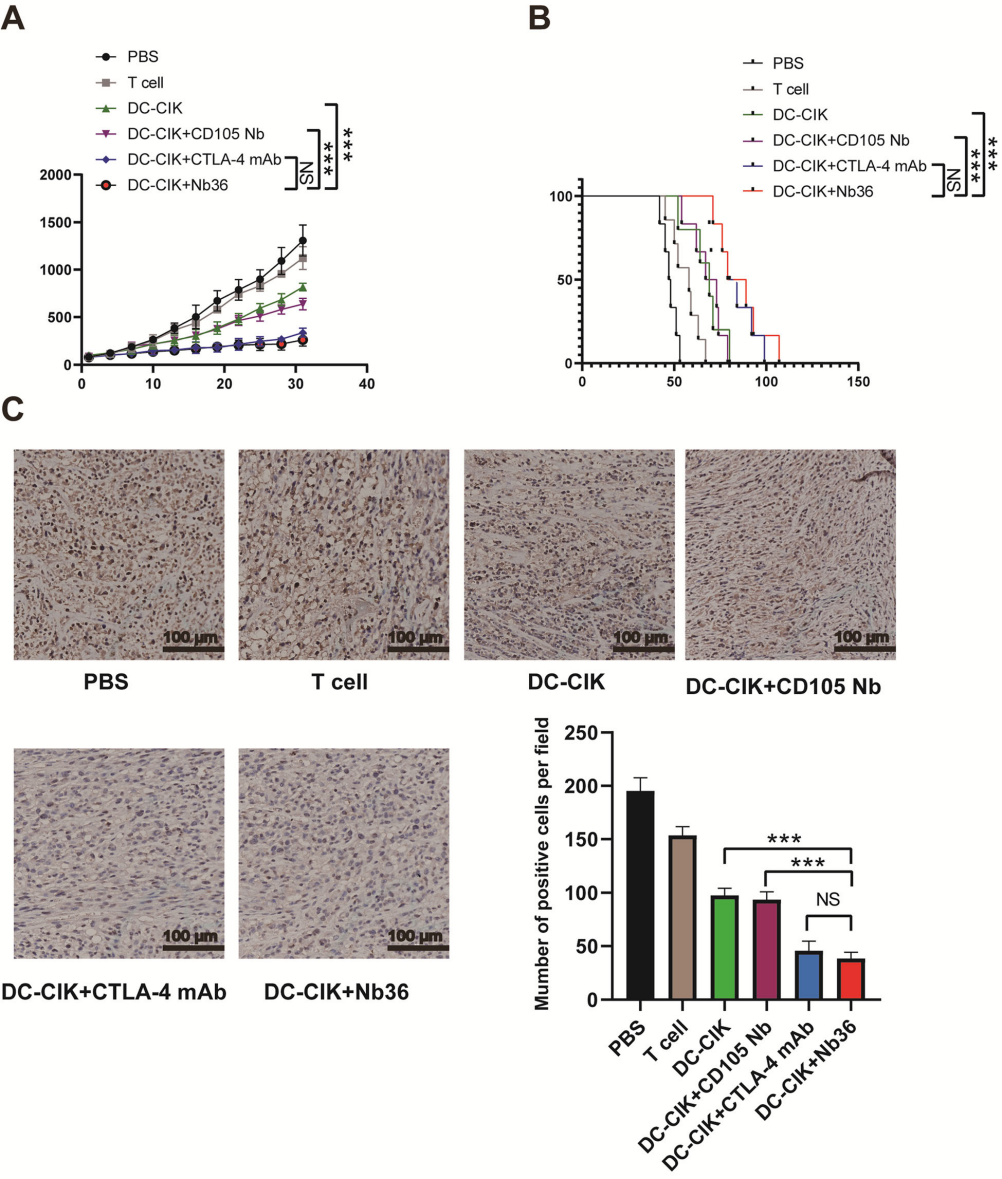
In vivo antitumor activities of Nb36-treated DC-CIK cells in the established subcutaneous human tumor xenografts. **A** The tumor growth curves. Nb36-treated DC-CIK cells significantly reduced tumorvolume. *n* = 6, *** *P* < 0.001. **B** The survival of tumor-bearing mice. Nb36-treated DC-CIK cells prolonged the survival of tumor-bearing mice. *n* = 6, *** *P* < 0.001. **C** Quantitative analysis of KI-67 expression. Data are representative images (magnification × 400) or expressed as the mean ± SD from 5 randomly selected fields ofthin tumor sections. *n* = 6, ***P < 0.001

We examined the effects of Nb36-treated DC-CIK cells in vivo. It was found that treatment with DC-CIK Cells+Nb36 was able to significantlyreduce the volume and weight of the tumors in mice, and that it prolonged the survival of tumor-bearing mice when compared with PBS, T-cells, DC-CIK and DC-CIK + CD105 Nb groups (Fig. 5A-B). Similarly, the immunohistochemical analysis of the xenograft tumor tissue revealed that the number of anti-Ki67 cells in the mice receiving Nb36-treated DC-CIK cells wassignificantly lowerthan in any of the other groups (Fig. 5C). These data therefore indicate that treatment with Nb36-treated DC-CIK cells inhibits the growth of implanted tumors in mice.

## Discussion

Compared with traditional methods, tumor immunotherapy has become a promising strategy for the treatment of malignant tumors. Numerous clinical studies have shown that anactivation of the anti-tumor effector cells for adoptive introduction into the patient could improve the prognosis [20, 21]. Thisis very important for the CD3^+^CD8^+^cytotoxic T-cells subgroup, which isinduced by DCs in specific cellular immune responses. After induction, DC-CIK cells have the potential for both MHC-unrestricted and tumor-specific cytotoxicity [22]. Induction with anti-CTLA-4 monoclonal antibodieswas shown to promote DC-CIK cell proliferation and differentiation into CD3^+^CD56^+^ NK T-cells, which is the main effector of DC-CIK cells,providing synergistic antitumor effects by up-regulating the secretion of pro-inflammatory cytokine and decreasing the production of immunosuppressive cytokines in vivo [23].

The CTLA-4 pathway delivers inhibitory signals that negatively regulate effector T-cells to inhibit their activation. Several studies have revealed that the effects of DC-CIK cells arecombined with theeffects from the monoclonal antibodies targeting CTLA-4 [24]. However, the large molecular weight of monoclonal antibody drugs limits their penetration and concentration in solid tumors, which results inthe tumor treatment’s poor effectiveness. Moreover, the high costsinvolved prevents its widespread usage [25]. In order to overcome the above disadvantages, novel antibodies including single chain antibodies, genetically-engineered antibodies and nanobodies have been developed [26]. Specifically, and in view of the unique structural and molecular characteristics of Nb, Nb-targeting CTLA-4 may be an effective strategy to enhance the effect of DC-CIK cells.

Our study successfully obtained CIK cells from PBMCs, as was shown after the 12-day in vitro expansionwhereanti-CD3 antibodies, IFN-γ and PHA were sequentially added. Previous studies have shown that CIK cells have the dual properties of NK and T-cells [27], which is consistent with our research, as shown bythe positive expression of CD3 and CD56 immune markers. DCs are the most significant antigen-presenting cells that stimulate effector T-cells to enhance the immune response. We lysed all the tumor cells in order to generate tumor antigens to then induce DC maturation. Compared with the method of stimulating specific tumor antigens, this method can stimulate immunity against the entire tumor antigen, thereby inducing a more complete cytotoxicity [28]. We found that the proliferation activity of CIK cells was enhanced after DC co-cultivation, which becamemore pronounced as the CTLA-4 was blocked. Compared withthe control group, the levels of pro-inflammatory cytokines viz. TNF-α and IL-2, except IL-10, in the DC-CIK + Nb36 group’s culture medium,hadall increased following the co-culture with target cells. The in vivo treatment of Nb36-treated DC-CIK cells displayed an inhibited tumor growth and prolonged the survival of human tumor xenograft mice. The immunohistochemical analysis displayed a decreased number of cells with proliferative marker Ki67. Accordingly, our study showed that Nb36-treated DC-CIK cells effectively killed target cells both in vitro and in vivo. By binding to CTLA-4 on the surface of the effector lymphocytes, Nb36 was able to increase the activation of DC-CIK cells, with stronger cytotoxicity and anti-tumor effects. This CTLA-4 blocking strategy is expected to be widely used as an alternative for anti-tumor therapy due to the high prices of anti-CTLA-4 mAb drugs. In ournext study, weaim tolook at improvingNb36’santi-tumor activity by adding FC fragments and carrying nanomaterials, as well as trying to apply Nb36 to anti-tumor therapy in vivo.

## Conclusions

This isthe first study to report on the application of nanobodies as CTLA-4 blockers to enhance the efficacy of DC-CIK cells. This preclinical study presents a new strategy and a promising prospect for the immunotherapy of malignant tumors. The results encourage further research on the efficacy of patient-derived DC-CIK cells combined with anti CTLA-4 nanobodiesforthe treatment of malignant tumors.

## Data Availability

The datasets used and/or analyzed during the current study areavailable from the corresponding author on reasonable request.

## Abbreviations

Nb: Nanobody
DC-CIK: Cytokine-induced killer cells which were induced with tumor antigen-pulsed dendritic cells
CTLA-4: Cytotoxic T lymphocyte-associated antigen-4
PBMC: Peripheral blood mononuclear cells
